# Multi-omics analyses reveal the specific changes in gut metagenome and serum metabolome of patients with polycystic ovary syndrome

**DOI:** 10.3389/fmicb.2022.1017147

**Published:** 2022-10-19

**Authors:** Zhandong Yang, Huijiao Fu, Huihui Su, Xuzi Cai, Yan Wang, Yanjun Hong, Jing Hu, Zhiyong Xie, Xuefeng Wang

**Affiliations:** ^1^Department of Obstetrics and Gynecology, The Third Affiliated Hospital of Southern Medical University, Guangzhou, China; ^2^School of Pharmaceutical Sciences (Shenzhen), Sun Yat-Sen University, Guangzhou, China; ^3^Department of Gastroenterology, First Affiliated Hospital of Guangzhou Medical University, Guangzhou, China; ^4^Institute of Biological and Medical Engineering, Guangdong Academy of Sciences, Guangzhou, China; ^5^Guangdong Engineering Research Center for Sugar Technology, Guangzhou, China; ^6^Department of Obstetrics and Gynecology, Jianli Fourth People’s Hospital, Jingzhou, China

**Keywords:** polycystic ovary syndrome, gut microbiota, shotgun metagenomics, serum metabolome, untargeted metabolomics, fecal microbiota transplantation

## Abstract

**Objective:**

The purpose of this study was to investigate the specific alterations in gut microbiome and serum metabolome and their interactions in patients with polycystic ovary syndrome (PCOS).

**Methods:**

The stool samples from 32 PCOS patients and 18 healthy controls underwent the intestinal microbiome analysis using shotgun metagenomics sequencing approach. Serum metabolome was analyzed by ultrahigh performance liquid chromatography quadrupole time-of-flight mass spectrometry. An integrative network by combining metagenomics and metabolomics datasets was constructed to explore the possible interactions between gut microbiota and circulating metabolites in PCOS, which was further assessed by fecal microbiota transplantation (FMT) in a rat trial.

**Results:**

Fecal metagenomics identified 64 microbial strains significantly differing between PCOS and healthy subjects, half of which were enriched in patients. These changed species showed an ability to perturb host metabolic homeostasis (including insulin resistance and fatty acid metabolism) and inflammatory levels (such as PI3K/Akt/mTOR signaling pathways) by expressing sterol regulatory element-binding transcription factor-1, serine/threonine-protein kinase mTOR, and 3-oxoacyl-[acyl-cattier-protein] synthase III, possibly suggesting the potential mechanisms of gut microbiota underlying PCOS. By integrating multi-omics datasets, the panel comprising seven strains (*Achromobacter xylosoxidans*, *Pseudomonas* sp. M1, *Aquitalea pelogenes*, *Porphyrobacter* sp. HL-46, *Vibrio fortis*, *Leisingera* sp. ANG-Vp, and *Sinorhizobium meliloti*) and three metabolites [ganglioside GM3 (d18:0/16:0), ceramide (d16:2/22:0), and 3Z,6Z,9Z-pentacosatriene] showed the highest predictivity of PCOS (AUC: 1.0) with sensitivity of 0.97 and specificity of 1.0. Moreover, the intestinal microbiome modifications by FMT were demonstrated to regulate PCOS phenotypes including metabolic variables and reproductive hormones.

**Conclusion:**

Our findings revealed key microbial and metabolite features and their interactions underlying PCOS by integrating multi-omics approaches, which may provide novel insights into discovering clinical diagnostic biomarkers and developing efficient therapeutic strategies for PCOS.

## Introduction

Polycystic ovary syndrome (PCOS) is characterized by clinical and/or biochemical hyperandrogenism, chronic oligo- and/or anovulation, and polycystic ovary morphology (2004). Patients with PCOS generally have poor reproductive performance. Moreover, PCOS has been reported to cause a higher risk of long-term complications, including type 2 diabetes, cardiovascular disease (CVD), and ovarian and endometrial cancer (Barry et al., 2014; Azziz et al., 2016; Zhu et al., 2021). In parallel to ongoing epidemics of obesity, PCOS incidence is increasing worldwide, afflicting 5–20% of reproductive women (Azziz et al., 2016). By contrast, studies focusing on the etiology and intervention of PCOS seem stagnant. The pathogenesis of PCOS is always attributed to a consequence of multifactorial coaction (i.e., susceptible gene mutations, unhealthy lifestyle, and environmental conditions); its therapeutic approaches remain currently limited with lifestyle modifications (Escobar-Morreale, 2018).

Gut microbiota is a pivotal environmental factor influencing host wellness. In the past decade, it has been extensively documented that intestinal dysbacteriosis is involved in occurrence and development of many metabolic disorders, including obesity, insulin resistance, type 2 diabetes, CVD, and non-alcoholic fatty liver disease (NAFLD; Li et al., 2019; Fan and Pedersen, 2021). Given that PCOS is closely linked to the above diseases, several studies have attempted to explore the gut microbiome changes and their associations with PCOS. According to previous reports, the diversity of gut microbiota is reduced in patients with PCOS; Several specific bacterial species including Bacteroidetes and Firmicutes are found to have an abnormal abundance in PCOS, and these changes in gut microbiota reveal strong correlations with PCOS manifestations encompassing estradiol (E2), testosterone (T), luteinizing hormone (LH), and follicle-stimulating hormone (FSH; Torres et al., 2018; Zhou et al., 2020a; Dong et al., 2021).

To understand the relationships between PCOS and gut microbiota, a few studies employ fecal microbiota transplantation (FMT) and cohousing approaches, thus suggesting the modulative effects of gut microbiota on phenotypes of PCOS (Qi et al., 2019; Han et al., 2021; Ho et al., 2021). Subsequently, metabolomics profiling of fecal samples derived from PCOS patients is performed to investigate the molecular mechanisms underlying gut microbiota mediating disease progression (Rajska et al., 2020; Zhou et al., 2020b). Proverbially, the circulating metabolome directly reflects systemic metabolism related to pathological metabolic disturbance. Previous metabolomics studies also find that many perturbed circulating metabolites may be attributed to the intestinal microbiome alterations in PCOS, such as secondary bile acids (Rajska et al., 2020; Yang et al., 2022). A recent study showed that tempol attenuated PCOS possibly *via* modulating gut microbiota-serum metabolites interaction, suggesting that deciphering the associations between gut microbiota and circulating metabolites can aid better understanding the roles intestinal microbiome playing in PCOS development (Li et al., 2021). However, to our best knowledge, studies focusing on the impact of gut microbiome on the systemic metabolism in PCOS remain absent.

In the present study, multi-omics analyses integrating shotgun metagenomics sequencing and untargeted metabolomics profiling were performed to investigate the specific changes in intestinal metagenome and serum metabolome of subjects with PCOS. Metagenomics functionality modules were explored to elucidate the possible mechanisms underlying the impact of gut microbiome on systematic metabolism in PCOS. Through integration of clinical data and multi-omics (metagenome and metabolome) datasets, we built a biological network for identifying the key microbiota and metabolite features of PCOS, and predicted this disorder using them. Moreover, we transplanted fecal microbiota from patients and healthy controls into pseudo sterile and letrozole-induced PCOS rats, respectively, to further clarify the roles of the gut microbiology modifications in its pathogenesis, which may provide novel insights into developing diagnostic and management strategies for this disease.

## Materials and methods

### Study participants

This study was approved by the ZhuJiang Hospital of Southern Medical University Institutional Review Board (2018-FCK-002). Written informed consent was provided by each participant. In this study, the 2003 Rotterdam criteria were used to differentiate PCOS and healthy subjects (Rotterdam ESHRE/ASRM-Sponsored PCOS consensus workshop group, 2004), which required the presence of at least two of the following three symptoms: oligo- or an-ovulation, clinical and/or biochemical hyperandrogenism, and polycystic ovary morphology. Additionally, those suffering from (a) gastrointestinal diseases; (b) heart, liver, or kidney dysfunction; (c) using drugs that affect metabolomic profiles, insulin levels, or sex hormones within 3 months before recruitment; and (d) consuming probiotic products or antibiotics within 1 month before recruitment were excluded from the study. Finally, 32 patients with PCOS and 18 healthy individuals were included in this study. The height and weight of each participant was determined to calculate the body mass index (BMI = body weight/height^2^). Serum and fecal samples were collected once between the third and fifth day of the menstrual cycle in the maternity center at ZhuJiang Hospital of Southern Medical University and immediately stored at −80°C for further analysis. Serum samples were used to measure the levels of T, E2, dehydroepiandrosterone sulfate (DHEAS), sex hormone binding globulin (SHBG), free androgen index (FAI = T × 100 /SHBG), LH, FSH, the ratio of LH to FSH (LH/FSH), Anti-Mullerian hormone (AMH), prolactin (PRL), fasting glucose (FBG), fasting insulin (FSIns), homeostasis model assessment of insulin resistance (HOMA-IR = FBG × FSIns /22.5), total cholesterol (TCHO), triglyceride (TG), low-density lipoprotein (LDL), high-density lipoprotein (HDL) and C-reactive protein (CRP), and metabolomics profiling. Fecal samples were used for microbiome analyses.

### DNA extraction

A total of 250 mg fecal sample was used for DNA extraction using the PureLinkTM Stool Genomic DNA Kit (ThermoFisher Scientific Inc., Massachusetts, United States), following the manufacturer’s protocol. The DNA integrity and molecular size were detected using agarose gel electrophoresis, and DNA concentrations were quantified using Qubit® Fluorometer (ThermoFisher Scientific Inc., Massachusetts, United States).

### Metagenomic sequencing and analysis

Following the construction of the DNA libraries, all samples were sequenced from the 150-bp paired-end on the Illumina Hiseq™ 4000 platform. The adapter contamination and low-quality bases (*Q* < 20) were deleted from the raw sequencing reads. Reads belonging to human host DNA were removed based on the alignment in the human genome database using the Trimmomatic software. The high-quality reads were assembled with the SPAdes software and a reference gene catalog was generated, which contained the genes predicted from assembled contigs using the MetaGeneMark software. The gene profiles were obtained by aligning high-quality sequencing reads to the reference gene catalog, and the relative abundance profiles of microbes were calculated from the relative abundances of their respective genes. Gene counts were calculated by counting the number of genes that were determined in each sample. The functional characteristics were analyzed by annotating the nonredundant genes against the Kyoto Encyclopedia of Genes and Genomes (KEGG) database using BLAST, and the relative abundance of each KEGG Orthology (KO) was calculated from the relative abundance of all orthologous genes. Alpha diversity reflected by the Shannon–Wiener index (Shannon index) and Simpson index was assessed based on the gene profile of each sample, and Beta diversity dissimilarity between two groups was visually represented by the Bray_Curtis based principal coordinate analysis (PCoA). Differences between the two groups were calculated using the Wilcoxon rank-sum test adjusted with the Benjamini-Hochberg equation. Pearson’s calculation principle was applied to conduct the correlation analysis and calculate the probability and frequency of microbes in each sample.

### Serum untargeted metabolomic profiling

A total of 450 μl HPLC-grade acetonitrile was added to 50 μl serum sample, vortexed for 2 min, ultrasonicated for 2 min, and centrifuged for 10 min at 13,000 rpm at 4°C. The supernatant was collected for metabolomic profiling using ultra-high performance liquid chromatography-quadrupole time-of-flight mass spectrometry (UPLC-Q-TOF-MS). A total of 10 μl serum sample was mixed to prepare a quality control (QC) sample according to the above procedures. The QC sample was mainly used to monitor the system and column stability. Before the formal determination, 10 QC samples were injected to balance the system and column stability. Subsequently, one QC sample was injected after every six serum samples, and one blank sample for every 12 samples to monitor the instrument stability. All samples were randomly determined.

The chromatographic analysis was conducted using a Waters ACQUITY UPLC system equipped with a Q-TOF-MS (Waters Corporation, Milford, MA, United States) and an ACOUITY HSS T3 column (100 mm × 2.1 mm, 1.8 μm; Waters Corporation, Milford, MA, United States) under the positive and negative modes, respectively. The main parameters were as follows: column temperature, 40°C; mobile phase flow rate, 0.3 ml/min; injection volume, 2.0 μl in positive mode, and 3.0 μl in negative mode. The ratio of formic acid to water was 0.1% (v/v) for mobile phase A, and mobile phase B contained 0.1% (v/v) formic acid/mixture solvent (70% acetonitrile:30% isopropanol, v/v). The gradient parameters were as follows: 99.0% A at 0–2 min, 60% A at 2–5 min, 55% A at 5–7 min, 45% A at 7–14 min, 15% A at 14–20 min, 1% A at 20–23.1 min, and 99% A at 23.1 min. The automatic sampler was maintained at 4°C.

The mass spectrometry data were acquired using a SYNAPT G2-Si HDMS equipped with an electrospray ionization source (Waters Corporation, Milford, MA, United States). The main mass parameters were as follows: desolvation and cone gas, nitrogen gas; capillary voltage, 2.5 kV; nebulizer gas, 6.5 bar; cone voltage, 35.0 kV; cone gas flow rate, 40 L/h; desolvation gas temperature, 350°C; desolvation gas flow rate, 750 L/h; and source temperature, 120°C.

All chromatographic and MS data were collected using MassLynx V4.1 (Waters Corporation, Milford, MA, United States) and imported into the Progenesis QI V2.0 software (Waters Corporation, Milford, MA, United States) for background noise elimination and peak alignment. Each peak was annotated through the mapping of the mass-to-charge ratio and retention time against the reference information in a public database, such as METLIN,^1^ the human metabolome database,^2^ and Lipid Maps.^3^ Each metabolite was further characterized according to the accurate MS information acquired from MSE, DDA, and MS/MS modes.

Data pre-processing, including the elimination of peaks or samples with a missing value ratio over 50%, zero padding with the *k*-nearest Neighbor approach and normalization with the QC SVR (MetNormalizer), was conducted using the MetFlow Platform.^4^ Subsequently, multivariate statistical analysis was performed by constructing orthogonal partial least squares discriminant analysis (OPLS-DA) using the SIMCA V14.1 software (Umetrics, Sweden). The reliability of OPLS-DA models was validated by the permutation test (*n* = 200) and cross-validated ANOVA (CV-ANOVA). Furthermore, the differential metabolites between the two groups were screened according to the variable importance in the projection (VIP) > 1.5 in OPLS-DA, fold change >2.0, and *p* value adjusted by the BH method <0.05. Pathway analysis was performed using MetaboAnalyst 4.0^5^ based on the differential metabolites.

### Animal experiments

Sprague–Dawley (SD) female rats ranging from 5 to 6 weeks were obtained from the Guangdong Medical Laboratory Animal Center (Foshan, China) and kept under specific-pathogen-free conditions with a 12 h light/dark cycle in the Sun Yat-Sen University Laboratory Animal Center (Guangzhou, China). During the study, each rat had free access to food and water. Before the induction of the PCOS model, all animals suffered from a 7-day acclimatization period.

#### Animal experiment 1

The 16 SD rats were treated with broad-spectrum antibiotics (vancomycin 0.5 g/L, neomycin 1.0 g/L, metronidazole 1.0 g/L, and ampicillin 0.5 g/L) for 4 days, and subsequently washed out for another 3 days. These rats were averagely divided into two groups: control and fecal microbiota transplantation (FMT) groups. Each rat in the FMT group was orally treated with fecal microbiota from patients with PCOS (10^9^ CFU/rat), meanwhile, the control rats were orally administrated with sterile PBS once each day for 3 weeks. All animals were weighed every 7 days. From the 11th to 21st day, the oestrous cycles of all rats were determined once each day. At the end of this experiment, several parameters, including fasting blood glucose level (FBG), oral glucose tolerance test (OGTT), fasting serum insulin level (FSIns), and insulin tolerance test (ITT), were evaluated, and serum samples were collected. After sacrificing each rat, ovarian tissue samples were obtained for further analysis.

#### Animal experiment 2

The 24 SD female rats were averagely divided into four groups: control, letrozole, diane, and FMTT groups. The control rats were orally administered 0.2 ml PBS during the entire experiment while the other three groups were administrated letrozole (1.0 mg/kg·bw) for the first 3 weeks. Subsequently, the letrozole group was treated with 0.2 ml PBS and the diane group with Diane-35 (1.0 mg/kg·bw), which contains cyproterone acetate and ethinylestradiol, the first-line drug for PCOS treatment. The FMTT group was administrated with the fecal microbiota (10^9^ CFU/rat) of healthy donors for another 3 weeks. The oestrous cycle of each animal was measured once a day from the 32nd to 43rd day. Additionally, various indicators, including FBG, OGTT, FSIns, and ITT, were determined, and serum and ovarian tissue samples were collected after sacrificing the rats.

### Determination of oestrous cycle

Estrous cycle of each rat was determined according to a previous study (Kelley et al., 2016). The oestrous cycle stage of each rat was determined by the vaginal smears method at 10:00 am daily from the 11th to 21st day in animal experiment 1 and from the 32nd to 43rd day in animal experiment 2. Following Wright’s staining, the predominant cell type in vaginal smears was identified using an optical microscope.

### OGTT and ITT

Rats underwent fasting for 12 and 4 h before the OGTT and ITT, respectively. Glucose levels were determined by tail vein blood sampling using a ONETOUCH Ultra Easy glucose meter (Johnson & Johnson, New Jersey, United States). After the measurement of fasting glucose levels, the rats were orally administrated with D-glucose (2.0 g/kg·bw) for OGTT or insulin (1.0 IU/kg·bw) for ITT. The detection of glucose levels was performed at the following time points: 15, 30, 60, 90, and 120 min after the oral administration of D-glucose or insulin.

### Serum biochemical parameters

Serum sample of each rat was used for measuring FSIns, T, E2, LH, FSH, TCHO, TG, LDL, HDL, and CRP *via* ELISA kits (MAISHA Industries, Jiangsu, China), following the manufacturer’s instructions.

### Ovarian histopathological analysis

Ovarian histopathological examination and cystic follicle count were performed based on a previous study (Qi et al., 2019). Ovary tissues were quickly collected and fixed in 4% paraformaldehyde. Subsequently, a series of operations, such as dehydration, paraffin embedding, and segmentation, were performed. The sections were stained with hematoxylin and eosin for histopathological examination using a Pannoramic250 microscope (3DHISTECH Ltd., Budapest, Hungary).

### Statistical analyses

Statistical analyses were performed using the independent-sample *T* test, two-tailed Mann–Whitney U-test, or one-way ANOVA followed by Tukey’s post-hoc test. The Statistical Product and Service Solutions software (SPSS V24.0, Chicago, IL, United States) were used for all statistical analyses. *p* < 0.05 was considered statistically significant. Correlation analysis was performed using Pearson’s method. Performance of single/multi-omics dataset for predicting PCOS was examined by receiver operating characteristic (ROC) curves, and the panel integrating microbiota or/and metabolites was generated by binary logistic regression model. Data visualization was performed using R language packages or the OriginPro version 2021 software (OriginLab, MA, United States). Data are presented as the mean ± standard deviation.

## Results

### Clinical baseline characteristics of participants

Clinical characteristics of all subjects are summarized in Table 1. The reproductive hormones, including T, DHEAS, FAI, LH, LH/FSH, and AMH, were significantly higher in women with PCOS than in healthy controls (*p* < 0.01, Table 1). Metabolic variables, such as BMI, FBG, FSIns and HOMA-IR, were also markedly improved in PCOS (*p* < 0.0001, Table 1). By contrast, serum SHBG concentrations were significantly reduced in PCOS (*p* < 0.01, Table 1). Despite no statistical difference, there is an elevated trend in TCHO, TG, and LDL. Additionally, concentrations of the inflammatory cytokine CRP were significantly up-regulated in the PCOS group compared to the control group (*p* < 0.001, Table 1).

**Table 1:** Clinical baseline characteristics of all participants.

**Parameters**	**Control group (*n* = 18)**	**PCOS group (*n* = 32)**
Age (Years)	29.89 ± 3.36	29.34 ± 2.88
BMI	20.44 ± 2.20	23.46 ± 3.67^**^
T (μg/L)	0.44 ± 0.15	0.68 ± 0.30^*^
DHEAS (μg/dL)	190.30 ± 45.85	270.15 ± 83.75^**^
SHBG (nmol/L)	88.75 ± 21.08	48.00 ± 20.20^**^
FAI	0.57 ± 0.35	2.10 ± 2.74^**^
E2 (pmol/L)	235.83 ± 261.42	233.66 ± 129.57
LH (IU/L)	5.51 ± 1.92	10.75 ± 9.64^*^
FSH (IU/L)	7.28 ± 1.90	6.69 ± 1.66
LH/FSH	0.76 ± 0.24	1.57 ± 1.15^**^
AMH (μg/L)	3.19 ± 0.79	8.12 ± 4.60^***^
PRL (μg/L)	13.07 ± 4.59	13.02 ± 5.57
FBG (mmol/L)	4.63 ± 0.66	5.30 ± 0.34^**^
FSIns (mIU/L)	4.53 ± 2.20	9.70 ± 5.54^**^
HOMA-IR	0.92 ± 0.42	2.29 ± 1.35^**^
TCHO (mmol/L)	4.82 ± 0.79	5.17 ± 0.91
TG (mmol/L)	1.06 ± 0.86	1.11 ± 0.66
LDL (mmol/L)	2.65 ± 0.41	3.00 ± 0.78
HDL (mmol/L)	1.62 ± 0.34	1.48 ± 0.25
CRP (mg/L)	1.36 ± 0.42	3.01 ± 2.47^**^

BMI, body mass index; T, total testosterone; DHEAS, dehydroepiandrosterone sulfate; SHBG, sex hormone binding globulin; FAI, free androgen index (FAI = T × 100/SHBG); E2, estradiol; LH, luteinizing hormone; FSH, follicle-stimulating hormone; LH/FSH, the ratio of LH to FSH; AMH, Anti-Mullerian hormone; PRL, prolactin; FBG, fasting blood glucose; FSIns, fasting serum insulin; HOMA-IR, homeostasis model assessment of insulin resistance (HOMA-IR = FBG × FSIns/22.5); TCHO, total cholesterol; TG, triglyceride; LDL, low-density lipoprotein; HDL, high-density lipoprotein; CRP, and C-reactive protein. Data are presented as mean ± standard deviation. The difference between PCOS and healthy controls is compared by the independent-samples T test approach, and *p* < 0.05 is considered as statistical significance.

* *p* < 0.05;

** *p* < 0.01;

*** *p* < 0.001.

### Gut microbiota diversity and community composition in patients with PCOS

Both Shannon and Simpson indices in patients with PCOS were significantly lower than those of the healthy controls, suggesting that the gut microbiota α-diversity was reduced in this disorder (*p* < 0.01, Supplementary Figures S1A,B). PCoA, also called as β-diversity, represents the community similarity of microbial samples. In the present study, the Bray_Cruits based PCoA score plot illustrated a clear separation of PCOS individuals from healthy subjects, indicating the microbiota community composition differing between these two groups (Supplementary Figure S1C).

To identify the specific changed microbiota, we depicted the gut microbiome signature at different taxa levels from the phyla to genera. At the phylum level, the microbial phyla with the highest abundance were Firmicutes (68.06%), followed by Bacteroidetes (17.74%), Proteobacteria (7.48%), and Actinobacteria (5.74%). They together accounted for 99.02% of the total sequences (Supplementary Figure S2A). Among the phyla with over 0.05% of abundance, Actinobacteria were significantly elevated in PCOS (*p* < 0.05, Supplementary Figure S2B). At the genus level, 1,880 bacteria were annotated in all fecal samples. The top 30 genus microbiota in abundance were listed in Supplementary Figure S3A. Among the genera with over 0.05% of abundance, seven microbial genera were significantly enriched in PCOS, including *Blautia*, *Coprobacillus*, *Actinomyces*, *Pseudomonas*, *Enterococcus*, *Erysipelatoclostridium*, and *Gordonibacter* (*p* < 0.05, Supplementary Figure S3B). Meanwhile, six genera were depleted, such as *Faecalibacterium*, *Roseburia*, *Parabacteroides*, *Phascolarctobacterium*, *Odoribacter*, and *Paraprevotella* (*p* < 0.05, Supplementary Figure S3B).

### Identification of discriminatory microbial strains in subjects with PCOS

**Figure 1:**
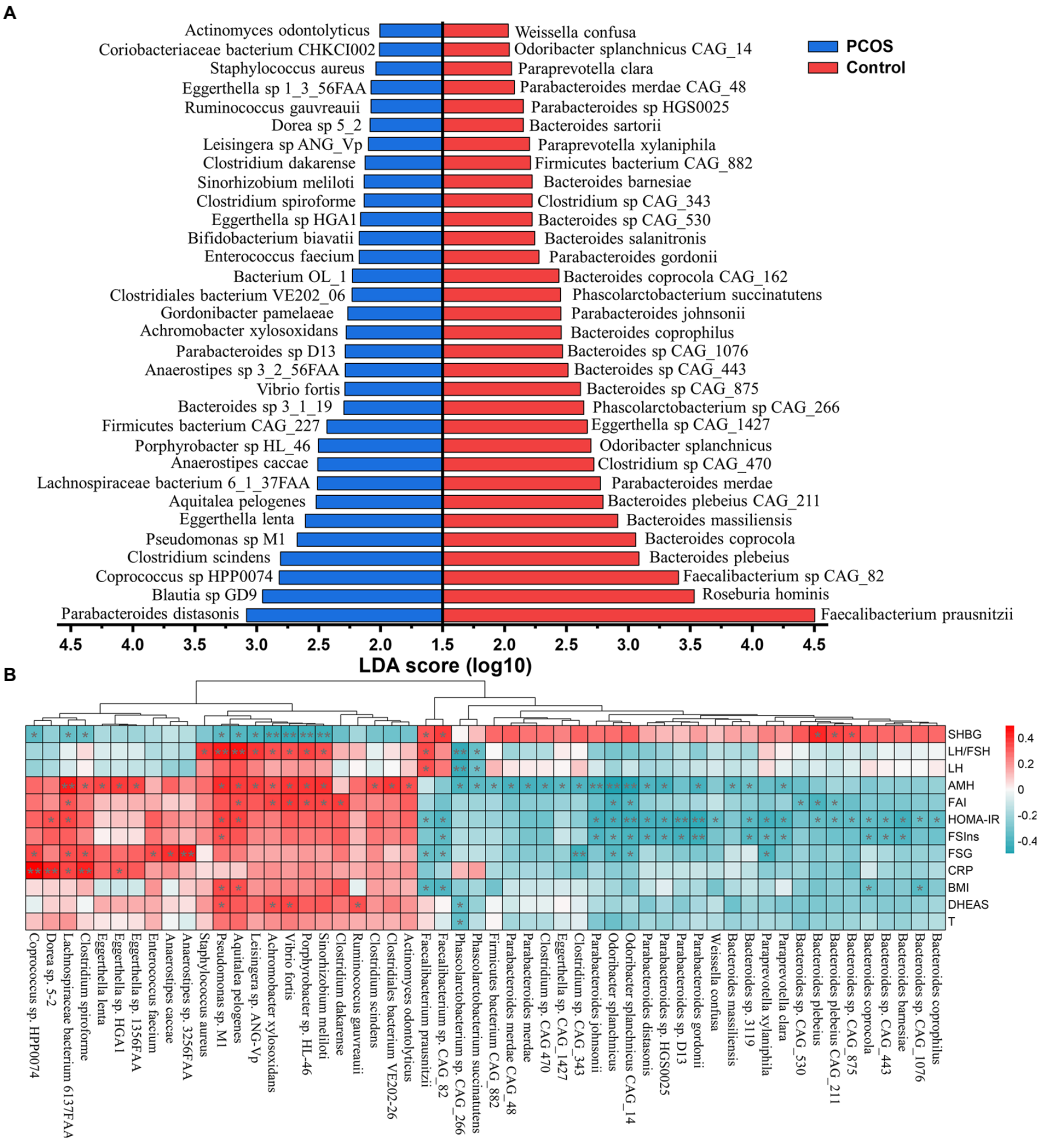
Identifying the significant changed gut microbial strains in PCOS. **(A)** Linear discrimination analysis (LDA) effect size (LEfSe) approach was used to identify the discriminatory microbial species with the LDA score (log10) ≥ 2.0 and the adjusted *p* < 0.05. **(B)** Correlation heatmap analysis between the differential strains and clinical variables in PCOS. SHBG, sex hormone binding globulin; LH, luteinizing hormone; LH/FSH, the ratio of LH to follicle-stimulating hormone (FSH); AMH, Anti-Mullerian hormone; FAI, free androgen index; FSIns, fasting serum insulin; HOMA-IR, homeostasis model assessment of insulin resistance; FSG, fasting serum glucose; CRP, C-reactive protein; BMI, body mass index; T, total testosterone; and DHEAS, dehydroepiandrosterone sulfate. Correlation coefficient between two indicators was calculated based on Pearson’s principle. Red and green colors represented positive and negative correlation, respectively, and the color depth showed the correlation coefficient size. ^*^ *p* < 0.05, ^**^ *p* < 0.01.

A large majority of the present studies focusing on gut microbiota of PCOS reveal changes at the genus levels, which may lead to heterogeneous and contradictory results because of the species diversity and complexity. To overcome this gap, we further characterized the composition signature of gut microbiota at the taxonomic species level. In the present study, a total of 10,461 strains were obtained in all fecal samples. Subsequently, the differential microbial species between the PCOS and healthy groups were identified by the LEfSe approach that assembles the statistical difference of each component with its impact. As shown in Figure 1A, there were 64 strains significantly differing between PCOS and healthy groups. Of which 32 species were significantly elevated in PCOS, including *Parabacteroides distasonis*, *Clostridium scindens*, *Eggerthella lenta*, *Aquitalea pelogenes*, *Anaerostipes caccae*, *Vibrio fortis*, *Gordonibacter pamelaeae*, *Clostridium spiroforme*, and *Staphylococcus aureus* (Figure 1A). Most of these species were pro-inflammatory or opportunistic pathogens. By contrast, the remaining half were depleted in PCOS, such as *Faecalibacterium prausnitzii*, *Roseburia hominis*, *Bacteroides plebeius*, *Bacteroides coprocola*, *Bacteroides massiliensis*, *Parabacteroides merdae*, *Odoribacter splanchnicus*, *Parabacteroides johnspnii*, and *Phascolarctobacterium succinatutens* (Figure 1A). Many of them have been reported to improve metabolic disorders including obesity, type 2 diabetes, and NAFLD.

The distribution of these differential microbial strains in each sample and their taxonomic attribution were illustrated in the heatmap (Supplementary Figure S4). These 64 differential species were mainly derived from four microbial phyla: Bacteroidetes (38.46%), Firmicutes (36.92%), Actinobacteria (12.31%), and Proteobacteria (10.77%). Notably, almost all species from the genus *Bacteroides*, except for *Bacteroides* sp. 3_1_19, were inhibited in PCOS (Supplementary Figure S4). Conversely, all seven strains (*Pseudomonas* sp. M1, *Aquitalea pelogenes*, *Vibrio frotis*, *Sinorhizobium meliloti*, *Achromobacter xylosoxidans*, *Leisingera* sp. ANG-Vp, and *Porphyrobacter* sp. HL-46) attributing to the phylum Proteobacteria were the main contributors in PCOS (Supplementary Figure S4).

To identify the gut microbiota closely associated with PCOS, a correlation analysis between the differential microbial species and clinical manifestations was performed. As shown in Figure 1B, these strains including *Pseudomonas* sp. M1, *A. pelogenes*, *V. frotis*, *S. meliloti*, *A. xylosoxidans*, *Leisingera* sp. ANG-Vp, and *Porphyrobacter* sp. HL-46 mainly had strong correlation with the reproductive hormones, such as LH, LH/FSH, AMH, FAI, and DHEAS (*p* < 0.05). Besides, they were significantly negatively correlated with SHBG that is also called as testosterone-estradiol binding globulin and plays a pivotal role in the conversion of T to E2 (*p* < 0.05). The microorganisms depleted in PCOS, including *F. rausnitzii*, *Faecalibacterium* sp. CAG_82, *P. johnsonnii*, and *O. splanchnicus* and the species from the phylum Bacteroidetes basically revealed strong association with metabolic variables like FSIns and HOMA-IR (*p* < 0.05, Figure 1B).

### Functional characteristics of gut microbiome in PCOS patients

To obtain more in-depth knowledge of the molecular mechanisms by which gut microbiota modulating PCOS pathogenesis, we performed the functionality analysis of intestinal microbiome using the metagenomic sequences. In the present study, 383 microbial metabolic pathways were annotated in the KEGG databases. Of these, 35 metabolic functions significantly differed between PCOS and healthy controls (Supplementary Table S1). Insulin resistance and insulin signaling pathway were the most major pathways affected by gut microbiome changes in PCOS (Supplementary Table S1). Additionally, it was observed that multiple pro-inflammatory signaling pathways, including mTOR, ErbB, JAK–STAT, PI3K-Akt, and MAPK signaling pathways, were significantly enriched in the PCOS group (Supplementary Table S1). By contrast, these metabolic pathways related to amino acids (phenylalanine, tyrosine, and tryptophan), vitamins (biotin, folate, and riboflavin), and antioxidants (porphyrin and chlorophyll) were markedly suppressed in patients with PCOS (Supplementary Table S1).

**Figure 2:**
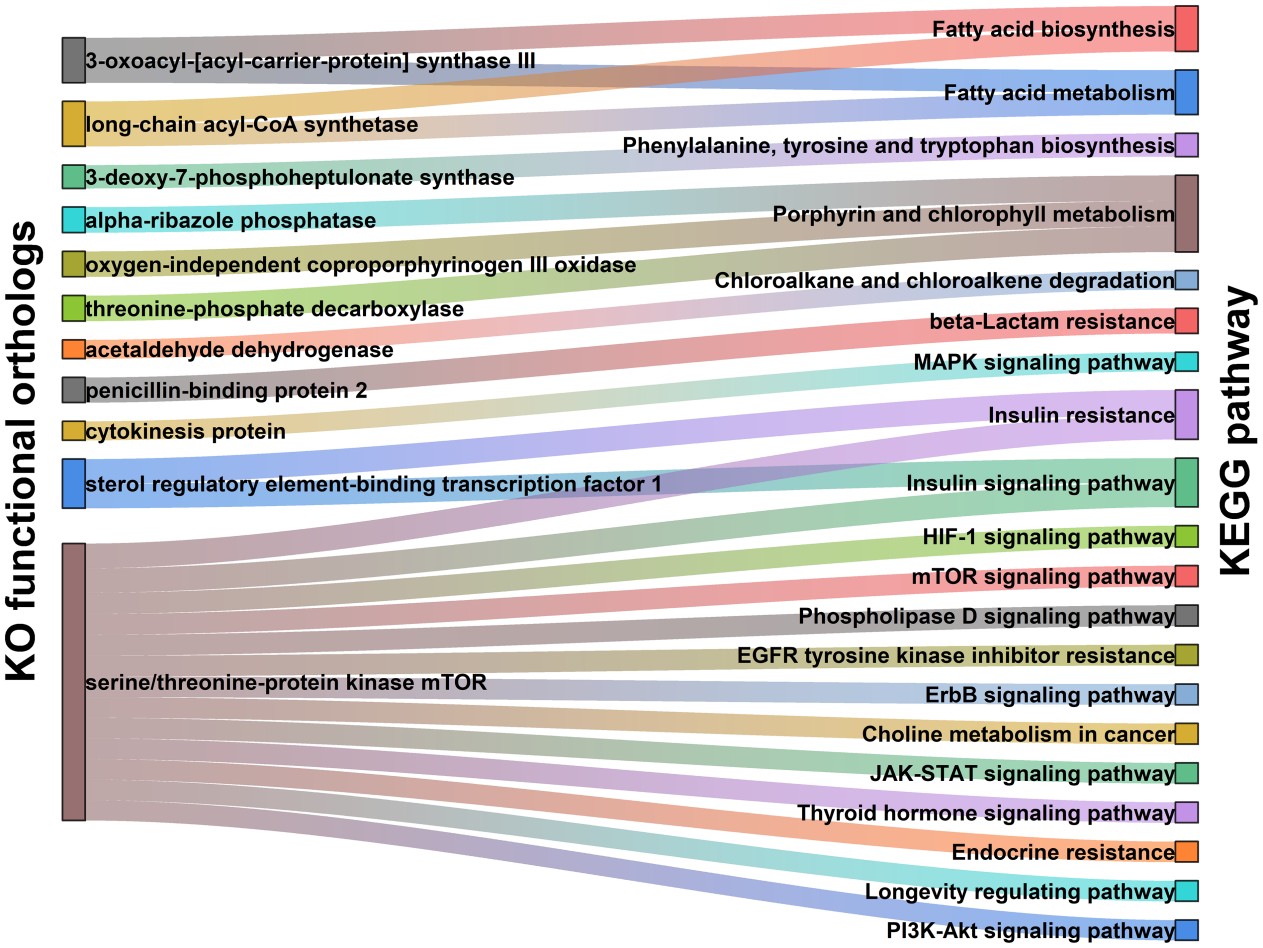
Functional module network of the differential microbiota in PCOS, including Kyoto Encyclopedia of Genes and Genomes (KEGG) Orthology (KO) functional orthologs (Left) and KEGG pathway (Right). Two nodes are connected when they show the direct interaction in KEGG database. The line width stands for the abundance of each metabolic pathway.

To exploit the precise roles of gut microbiota in actuating the above metabolic pathways, we further investigated molecular functions of the gut microbiome in PCOS. As shown in Supplementary Table S2; Figure 2, expression of sterol regulatory element-binding transcription factor 1 (SREBF1) that is involved in insulin resistance and insulin signaling pathway was significantly upregulated in PCOS compared to healthy controls (*p* < 0.001). Serine/threonine-protein kinase (MTOR) related to the mTOR, PI3K-Akt, JAK–STAT, and insulin signaling pathways also revealed an over-expression in PCOS (*p* < 0.001, Supplementary Table S2; Figure 2). In porphyrin and chlorophyll metabolism, the functional modules including alpha-ribazole phosphatase (phpB), oxygen-independent coproporphyrinogen III oxidase (hemZ), and threonine-phosphate decarboxylase (cobD) were significantly abolished in PCOS (*p* < 0.05, Supplementary Table S2; Figure 2).

### Identification of serum metabolome signatures in subjects with PCOS

It is well known that gut microbiota plays a major role in host metabolism. However, the direct evidence of intestinal flora regulating host metabolism in PCOS remains absent. In the present study, we undertook an untargeted metabolomics profiling using UPLC-Q-TOF-MS to depict the serum metabolome signatures and their association with gut microbiota in PCOS. As shown in Supplementary Figures S5A–D, the representative base peak chromatogram revealed clear differences between PCOS and healthy subjects under both positive and negative ion modes. Additionally, the unsupervised PCA score plots also showed the separation trends of patients with PCOS from healthy participants (Supplementary Figure S5E), indicating serum metabolite composition differing between the two groups.

**Figure 3:**
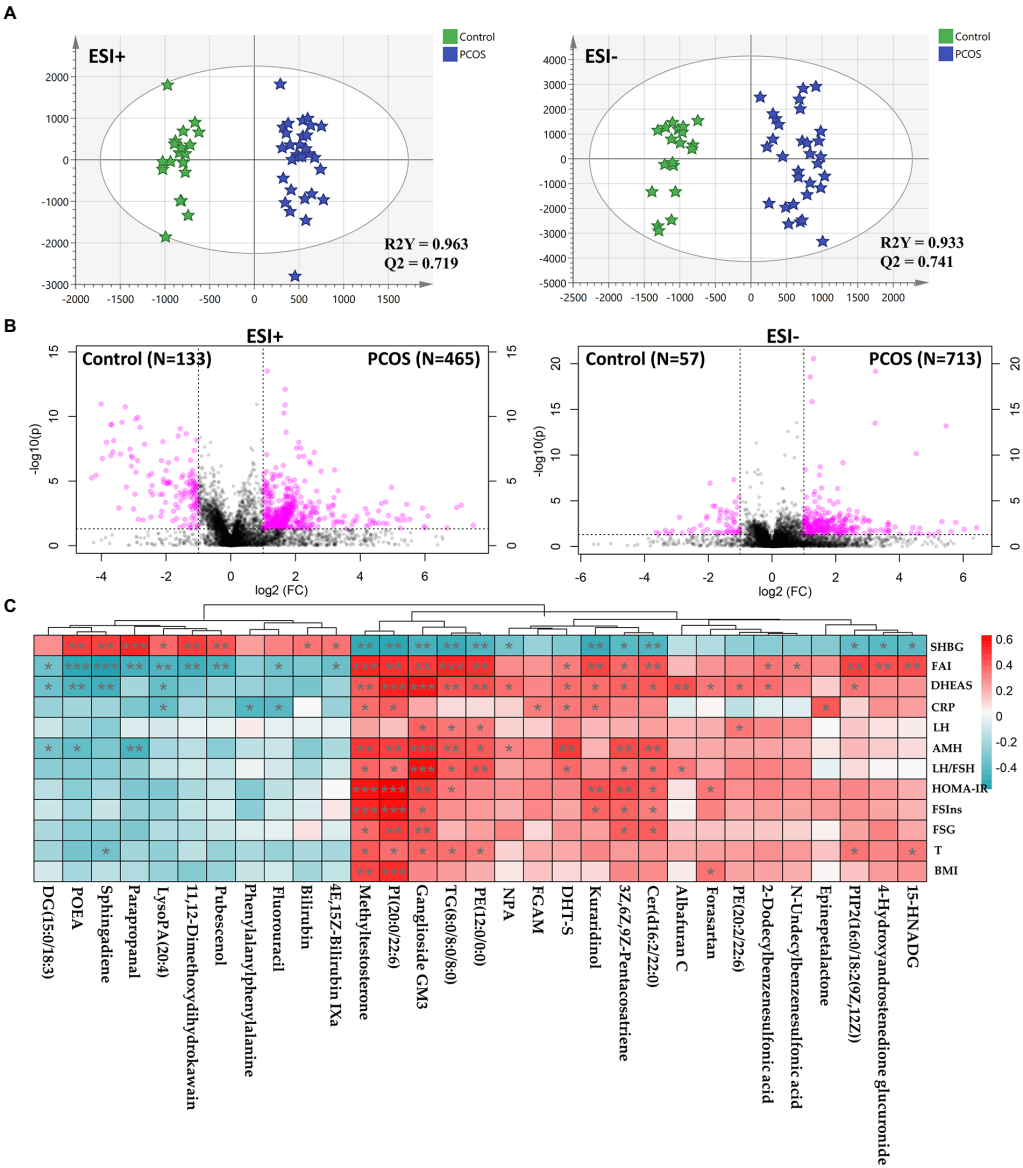
Serum metabolomics profiling underlying PCOS. **(A)** Orthogonal partial least-squares discrimination analysis (OPLS-DA) score plots constructed using untargeted metabolomics data. **(B)** Volcano plots used to screen differential compounds between PCOS and healthy subjects. **(C)** Correlation heatmap analysis between differential metabolites and clinical indicators in PCOS. DG, diacylglycerol; POEA, palmitoleoyl ethanolamd; LysoPA, lysophosphatidic acid; DHT-S, 5α-Dihydrotestosterone sulfate; FGAM, 5′-Phosphoribosyl-N-formylglycinamidine; Cer, ceramide; PI, phosphatidylinositols; TG, triglyceride; PE, phosphatidylethanolamine; PIP2, phosphatidylinositol diphosphate; 15-HNADG, 15-Hydroxynorandrostene-3,17-dione glucuronide; NPA, N-[2-(3,4-dimethoxyphenyl)ethyl]-3-[4-methoxy-3-(sulfooxy)phenyl]propanimidic acid; SHBG, sex hormone binding globulin; LH, luteinizing hormone; LH/FSH, the ratio of LH to follicle-stimulating hormone (FSH); AMH, Anti-Mullerian hormone; T, total testosterone; FSIns, fasting serum insulin; HOMA-IR, homeostasis model assessment of insulin resistance; BMI, body mass index; FSG, fasting serum glucose; CRP, C-reactive protein; DHEAS, dehydroepiandrosterone sulfate; and FAI, free androgen index. Correlation coefficient between two parameters was calculated based on Pearson’s approach. ^*^p < 0.05, ^**^p < 0.01, and ^***^p < 0.001.

To identify the difference, a supervised OPLS-DA model was constructed using the untargeted metabolomics data. As illustrated in Figure 3A, OPLS-DA score plots showed a significant separation of PCOS subjects from healthy controls, whose reliability was validated by permutation tests and CV-ANOVA *p* values (Supplementary Figure S5F). By integrating fold change and *p* value of each component, there were 1,368 compounds differing between the PCOS and healthy groups, including 598 and 770 in positive and negative ion modes, respectively (Figure 3B). Furthermore, we identified 35 differential metabolites between the two groups, most of which were phospholipids and hormones (Supplementary Table S3). There were 21 metabolites that were significantly upregulated in PCOS, including ganglioside GM3 (d18:0/16:0), methyltestosterone, and 5α-dihydrotestosterone (DHT-S; Supplementary Table S3). By contrast, the remaining 14 compounds were markedly downregulated in PCOS, such as sphingosine, bilirubin, and 4E,15Z-bilirubin IXa (Supplementary Table S3).

We subsequently investigated the correlation between differential metabolites and clinical features. As shown in Figure 3C, a large majority of metabolites revealed association with SHBG, FAI, and DHEAS. Of note, three compounds [GM3, PI(20:0/22:6), and methyltestosterone] had strong correlation with almost all PCOS-related phenotypes, including negatively associated with SHBG, and positively correlated with T, FAI, DHEAS, AMH, and LH/FSH (*p* < 0.05, Figure 3C). Two metabolites [Cer(d16:2/22:0) and 3Z,6Z,9Z-Pentacosatriene] were positively associated DHEAS, FAI, AMH, LH/FSH. FSG, FSIns, and HOMA-IR, whereas negatively correlated with SHBG (*p* < 0.05, Figure 3C). Enrichment analysis was performed to explore the effect of the changes in serum metabolites on its metabolome. In the present study, there were seven metabolic pathways enriched, including pentose and glucuronate interconversions, sphingolipid metabolism, porphyrin and chlorophyll metabolism, glycerophospholipid metabolism, and steroid biosynthesis (Supplementary Figure S6; Supplementary Table S4). Notably, several metabolic dysfunctions simultaneously occurred in both gut microbiome and serum metabolome in PCOS (Supplementary Figure S6; Supplementary Table S2), such as porphyrin and chlorophyll metabolism.

### Integrative analysis of multi-omics data and its prediction of PCOS

**Figure 4:**
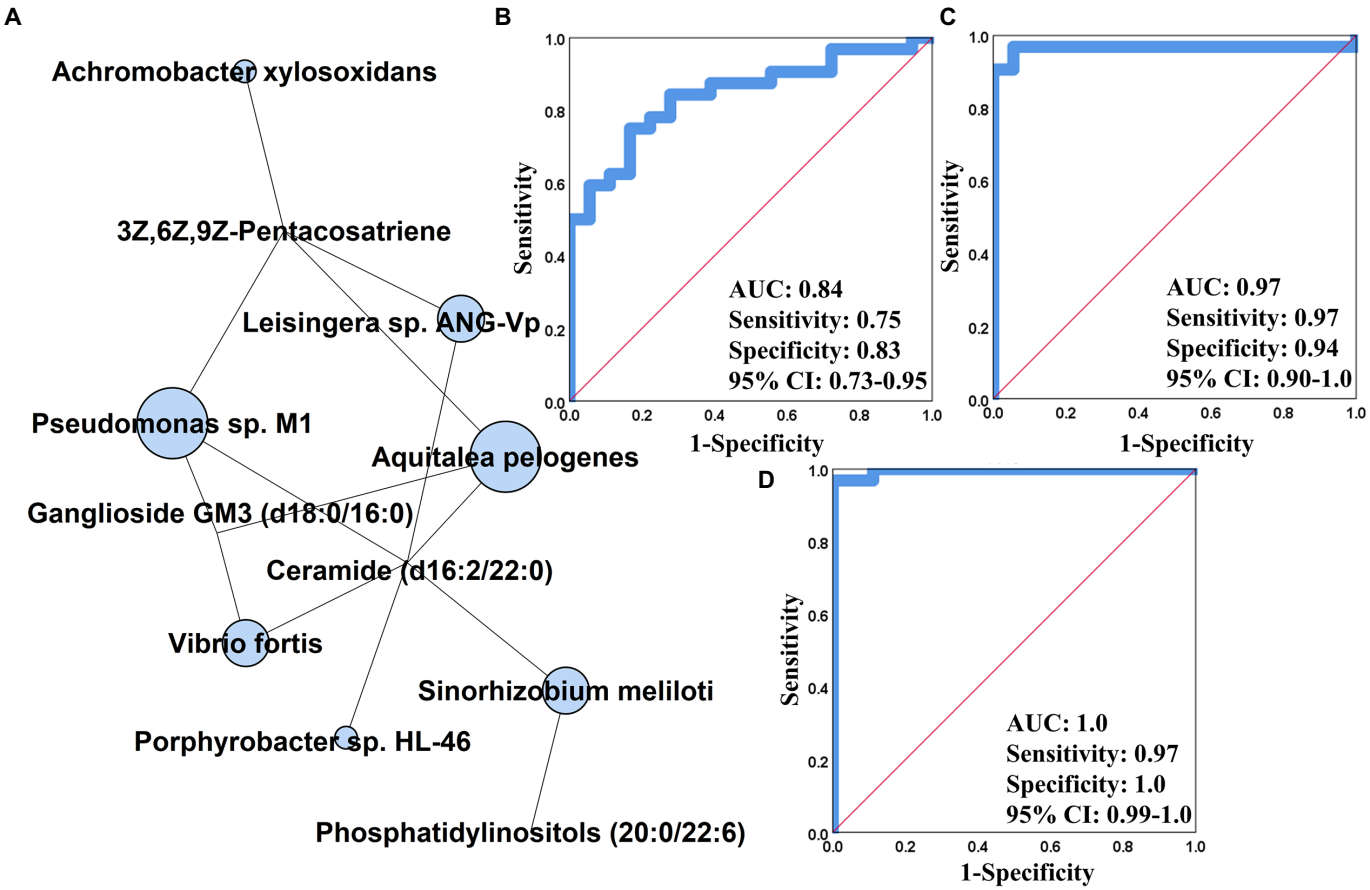
Integrative analysis using multi-omics data in PCOS. **(A)** Gut metagenomics and serum metabolomics-centric correlation network. The receiver operator characteristic curve (ROC) analysis of gut microbiome **(B)**, serum metabolome **(C)**, and gut microbiota-serum metabolite-based panel **(D)** constructed based on binary logistic regression model. AUC, the area under the curve; CI, confidence interval.

We generated an integrative multi-omics correlation heatmap for revealing the influence of gut microbiome on serum metabolome. As shown in Supplementary Figure S7, 46 of 64 strains were significantly associated with at least one of serum metabolites, and these microbial species were clustered into four groups. The cluster 1 comprising *R. hominis*, *F. prausnizii*, *B. salanitronis*, *B. massuliensis*, etc. mainly revealed correlation with the compounds depleted in PCOS (Supplementary Figure S7). The cluster 2 involving *P. succinatutens*, *O. splanchnicus*, *P. gordonii*, etc. was basically associated with the metabolites enriched in PCOS (Supplementary Figure S7). The cluster 3 was composed of eight strains (*A. odontolyticus*, *Pseudomonas* sp. M1, *A. pelogenes*, *A. xylosoxidans*, *Leisingera* sp. ANC-Vp, *V. fortis*, *Porphyrobacter* sp. HL-46, and *S. meliloti*), and showed significant positive and negative association with a large majority of serum metabolites (Supplementary Figure S7). Of note, the seven microbial species derived from the phyla Proteobacteria, including *Pseudomonas* sp. M1, *A. pelogenes*, *A. xylosoxidans*, *Leisingera* sp. ANC-Vp, *V. fortis*, *Porphyrobacter* sp. HL-46, and *S. meliloti*, were ambitiously positively correlated with ganglioside GM3, PI(20:0/22:6), Cer(d16:2/22:0), and 3Z,6Z,9Z-Pentacosatriene (|*r*| > 0.5, *p* < 0.01, Supplementary Figure S7). By constructing an interactive multi-omics correlation network, it was found that ganglioside GM3, Cer(d16:2/22:0), and 3Z,6Z,9Z-Pentacosatriene possibly played a central role in the influence of gut microbiome on serum metabolome in PCOS (Figure 4A). Furthermore, we performed the receiver operator characteristic curve (ROC) analyses using single/multi-omics data for discovering the key features distinguishing PCOS subjects from healthy controls. The microbiota-based panel comprising *Pseudomonas* sp. M1, *A. pelogenes*, *A. xylosoxidans*, *Leisingera* sp. ANC-Vp, *V. fortis*, *Porphyrobacter* sp. HL-46, and *S. meliloti* showed the excellent predictivity of PCOS with AUC of 0.84, sensitivity of 0.75 and specificity of 0.83 (Figure 4B). The metabolite-based panel composed of ganglioside GM3, Cer(d16:2/22:0) and 3Z,6Z,9Z-Pentacosatriene had an AUC of 0.97 with sensitivity of 0.97 and specificity of 0.94 (Figure 4C). By integrating the multi-omics datasets, the gut microbiome-serum metabolome panel yielded the highest prediction of PCOS (AUC: 1.0) with sensitivity of 0.97 and specificity of 1.0 (Figure 4D).

### Gut microbiota derived from patients induced PCOS features

**Figure 5:**
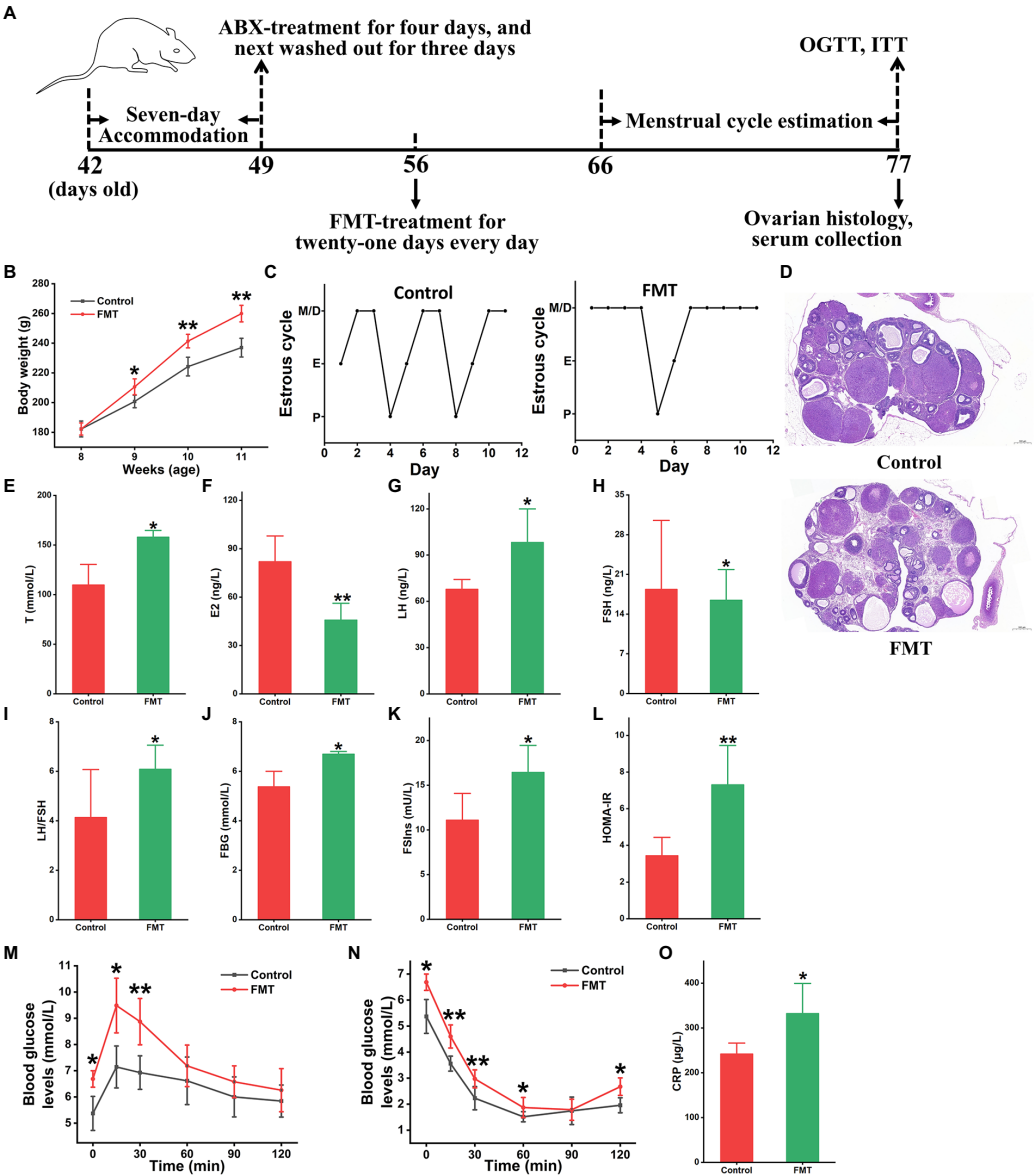
Gut microbiota from patients with PCOS induced disease-related phenotypes. **(A)** The schedule of this animal experiment; ABX, antibiotics (vancomycin, neomycin, metronidazole, and ampicillin); FMT, fecal microbiota transplantation; OGTT, oral glucose tolerance test; and ITT, insulin tolerance test. **(B)** Body weight change. **(C)** Menstrual cycle estimation. **(D)** Ovarian histopathology. Biochemical parameters include the levels of T **(E)**, E2 **(F)**, LH **(G)**, FSH **(H)**, LH/FSH **(I)**, FBG **(J)**, FSIns **(K)**, HOMA-IR **(L)**, OGTT **(M)**, ITT **(N)**, and CRP **(O)** in rats; T, testosterone; E2, estradiol; LH, luteinizing hormone; FSH, follicle-stimulating hormone; LH/FSH, the ratio of LH to FSH; FSG, fasting serum glucose; FSIns, fasting serum insulin; HOMA-IR, homeostasis model assessment of insulin resistance (HOMA-IR = FBG × FSIns/22.5); and CRP, C-reactive protein. *n* = eight rats in each group. Data are shown as mean ± standard deviation. Difference is compared by one-way ANOVA followed by Tukey’s post-hoc test. ^*^represents the comparison between FMT and control groups (^*^p < 0.05, ^**^p < 0.01).

To further confirm the impact of intestinal dysbacteriosis on circulating metabolism in PCOS, an FMT-conducted animal experiment was performed, in which fecal microbiota from patients with PCOS was transplanted into pseudo sterile rats (Figure 5A). As shown in Figure 5B, the recipient rats showed a distinct body-weight gain starting from the second week compared to that of the control rats (*p* < 0.05). The oestrous cycles of the rats receiving FMT stayed predominately in the metestrus and diestrus states (Figure 5C). Histopathological examination of ovarian tissues showed significant pathological features of PCOS in FMT rats, such as apoptosis and the loose arrangement of interstitial and granulosa cells, abnormal follicle development and increased cystic follicle numbers (Figure 5D). Moreover, the transplantation of fecal microbiota derived from patients caused a significant elevation in serum T, LH and LH/FSH levels and a reduction in E2 and FSH contents in the rats (*p* < 0.05, Figures 5E–I).

Additionally, the levels of metabolic variables (FBG, FSIns, and HOMA-IR) were significantly enhanced in the FMT-receiving rats (*p* < 0.05, Figures 5J–L). Moreover, the FMT group showed a significant tardive recovery in the OGTT and ITT experiments when compared to the control group (*p* < 0.05, Figures 5M,N). Serum concentrations of TG, TCHO, LDL, and HDL revealed slight differences between the FMT and control rats (Supplementary Figures S8A–D). Furthermore, fecal microbiota from patients with PCOS caused an increase in serum CRP concentrations in recipient rats (*p* < 0.05, Figure 5O).

### Restoring gut microbiome ameliorated PCOS phenotypes

**Figure 6:**
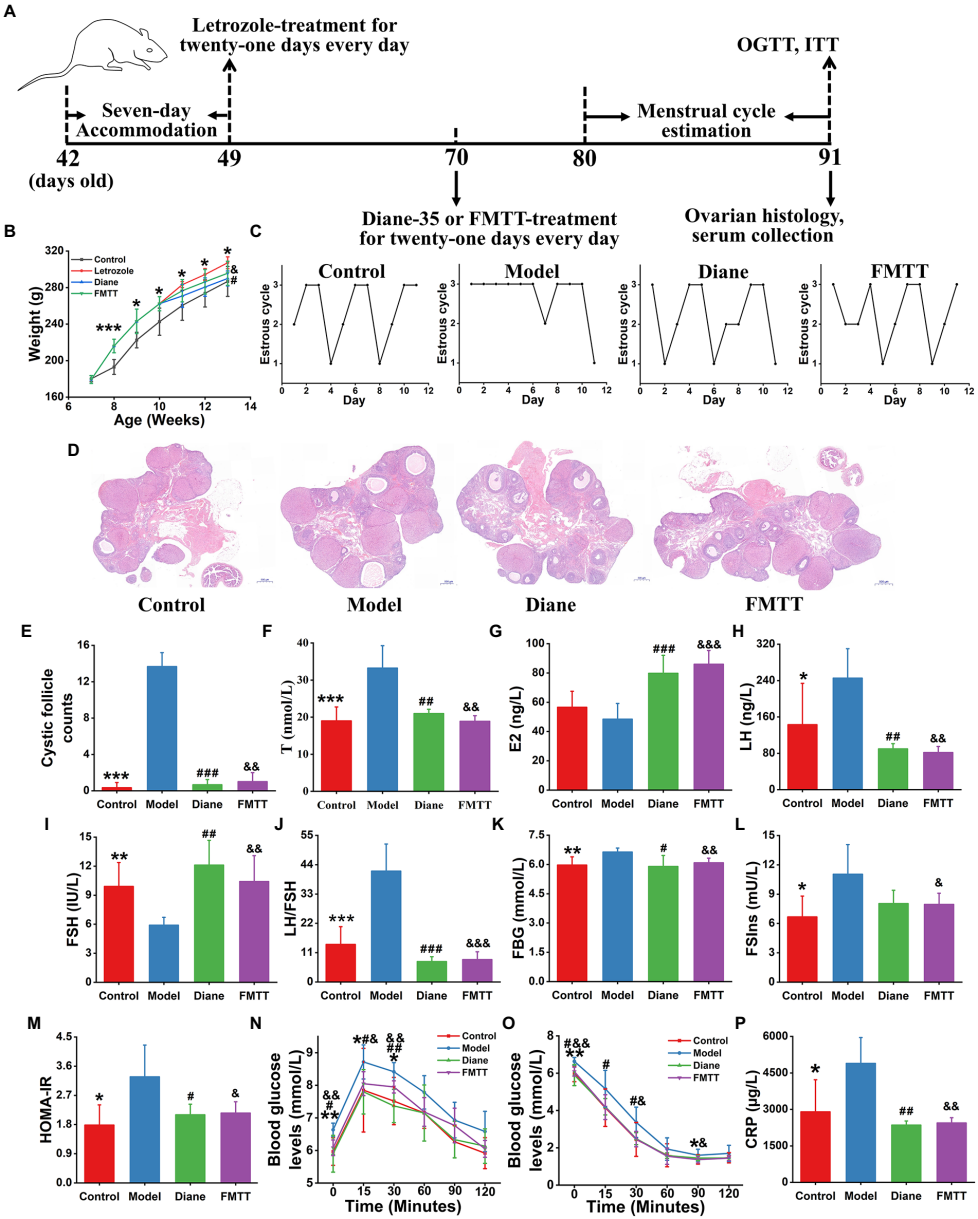
Healthy fecal microbiota attenuated letrozole-induced PCOS. **(A)** The schedule of this animal experiment; FMTT, the treatment by fecal microbiota transplantation; OGTT, oral glucose tolerance test; and ITT, insulin tolerance test. **(B)** Body weight change. **(C)** Menstrual cycle estimation. **(D)** Ovarian histopathology. **(E)** Cystic follicle counts. Biochemical parameters include the levels of T **(F)**, E2 **(G)**, LH **(H)**, FSH **(I)**, LH/FSH **(J)**, FBG **(K)**, FSIns **(L)**, HOMA-IR **(M)**, OGTT **(N)**, ITT **(O)**, and CRP **(P)** in rats; T, testosterone; E2, estradiol; LH, luteinizing hormone; FSH, follicle-stimulating hormone; LH/FSH, the ratio of LH to FSH; FSG, fasting serum glucose; FSIns, fasting serum insulin; HOMA-IR, homeostasis model assessment of insulin resistance (HOMA-IR = FBG × FSIns/22.5); and CRP, C-reactive protein. *n* = 6 rats in each group. Data are shown as mean ± standard deviation. Difference among four groups is compared by one-way ANOVA followed by Tukey’s post-hoc test. ^*^represents the comparison between control and PCOS model groups (^*^p < 0.05, ^**^p < 0.01, ^***^p < 0.001). # stands for the difference between diane-35 treatment and PCOS model groups (^#^*p* < 0.05, ^##^*p* < 0.01, ^###^*p* < 0.001). & indicates the difference between FMTT and PCOS model groups (^&^*p* < 0.05, ^&&^*p* < 0.01, ^&&&^*p* < 0.001).

We hypothesized that the reestablishment of gut microbiome could attenuate phenotypes of PCOS. To confirm this hypothesis, we performed another animal experiment, in which the gut microbiome composition and structure of the letrozole-induced PCOS rats was restored by the intervention of FMT from healthy donors (Figure 6). It was observed that the oestrous cycle, ovarian pathophysiology and reproductive hormones were significantly improved in the FMT-receiving rats (*p* < 0.05, Figure 6). Moreover, the restoration of gut microbiome also reversed serum levels of metabolic and inflammatory variables, such as FBG, FSIns, HOMA-IR, and CRP in the PCOS rats (*p* < 0.05, Figure 6).

## Discussion

**Figure 7:**
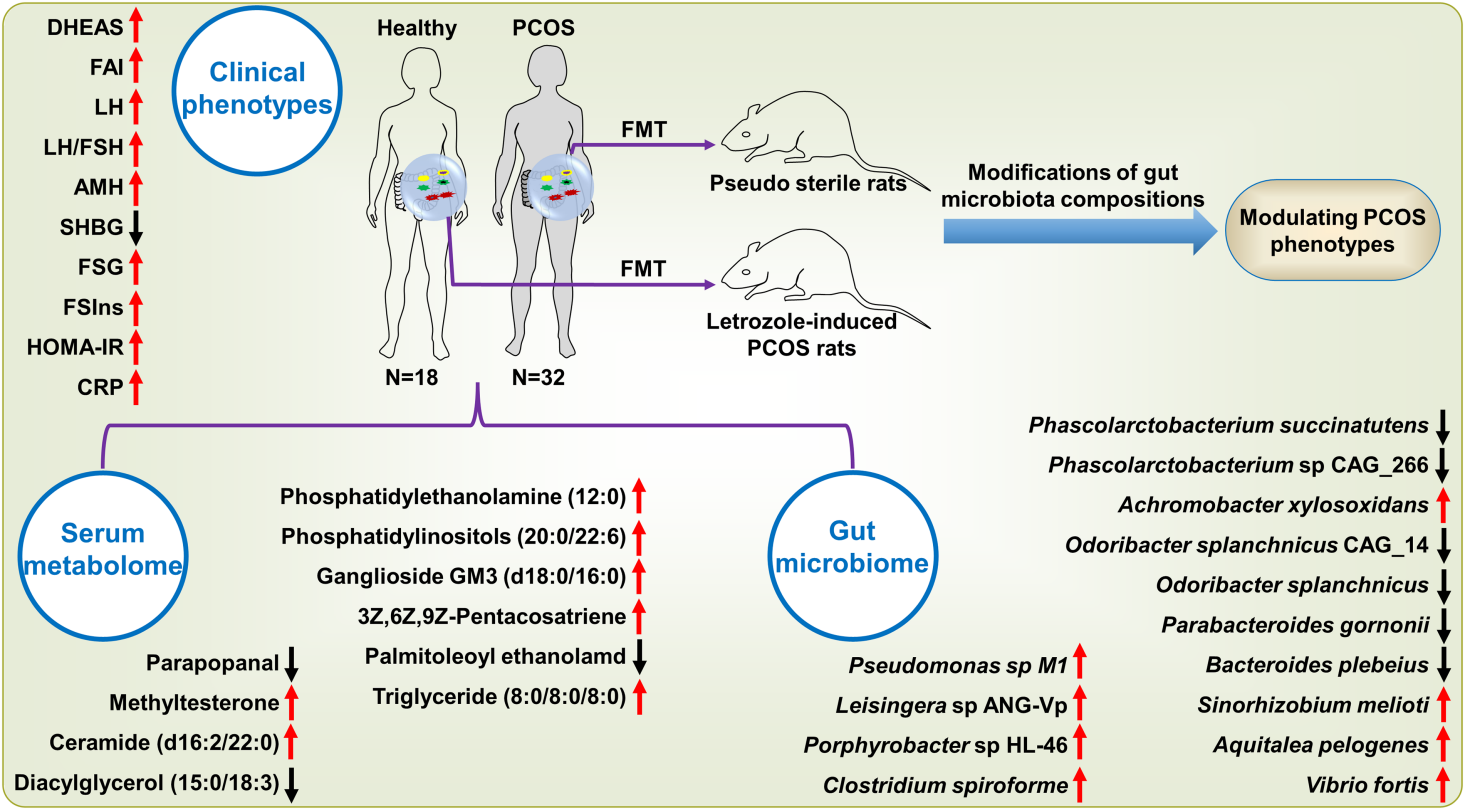
The key features in clinical phenotypes, gut microbiome and serum metabolome in PCOS. These variables are shown since they significantly differed between PCOS and healthy subjects and strongly associated with at least three analytes derived from other omics data. The arrow stands for the significant up-regulation or down-regulation of a variable in patients with PCOS as in comparison with healthy subjects. SHBG, sex hormone binding globulin; LH, luteinizing hormone; LH/FSH, the ratio of LH to follicle-stimulating hormone (FSH); AMH, Anti-Mullerian hormone; FSIns, fasting serum insulin; HOMA-IR, homeostasis model assessment of insulin resistance; FSG, fasting serum glucose; CRP, C-reactive protein; DHEAS, dehydroepiandrosterone sulfate; and FAI, free androgen index.

In the present study, we revealed the key findings of our multi-omics analyses in patients with PCOS (Figure 7). To discover the key microbial features associated with PCOS, we performed a shotgun metagenomics sequencing on feces from patients and observed the gut microbiota imbalance. By the LEfSe analysis, we identified 64 bacterial strains significantly differing between PCOS and healthy groups (Figure 1A). Half of these differential microbial species were enriched in PCOS, of which a large majority were pro-inflammatory and opportunistic pathogens, such as *C. scindens*, *Pseudomonas* sp. M1, *E. faecium*, and *S. aureus*. In previous microbiome studies using 16S rDNA amplicon sequencing approach, He et al. reported that the genus *Enterococcus* was significantly elevated in PCOS patients with insulin resistance (He and Li, 2021); Li et al. showed that abundance of *Staphylococcus* was clearly up-regulated, but reduced after intervention of tempol in PCOS (Li et al., 2021); Subsequently, a transcriptomics study showed that spleen tyrosine kinase, major histocompatibility complex class II (DR alpha and HLA-DRA), and interleukin 10 (IL-10), which are conducive to immunosuppression and inflammatory responses, were significantly over-expressed in the granulosa cells of patients with PCOS (Wang et al., 2020); Moreover, *S. aures* infection was reported to result in the elevation of HLA-DRA and IL-10 in granulosa cells. Therefore, *S. aures* was speculated to exacerbate PCOS by inducing HLA-DRA and IL-10 expressions in granulosa cells (Wang et al., 2020).

*Clostridium scindens* contains cortisol-induced operons (desABCD) that encode 20α-hydroxysteroid dehydrogenase (HSDH), steroid-17,20-desmolase/oxidase and corticosteroid transporter. The proteins, HSDH and steroid-17,20-desmolase/oxidase, catalyse glucocorticoids to 11β-hydroxyandrost-4-ene-3,17-dione (11β-OHA), and a dual carbon, the latter is introduced into the pentose and glucuronate pathway for catabolism (Ridlon et al., 2013). Therefore, *C. scindens* has the capacity of transferring glucocorticoids to androgen 11β-OHA *via* side-chain cleavage. Besides, *C. scindens* was also reported to synthesize androgen 1,4-androstanediene-3,11,17-trione from adrenocortical hormones (Ly et al., 2020). Both 11β-OHA and androgen 1,4-androstanediene-3,11,17-trione belonged to the family of androgen hormones. The species *Pseudomonas* sp. M1 attaching to the genus *Pseudomonas* was the most abundant in patients with PCOS (Figure 1A). A previous study demonstrates that the species derived from *Pseudomonas*, such as *P. aeruginosa*, express sulfatase, which catalyzes inactive DHEAS and DHT-S into active DHEA and DHT *via* desulfurization (Iannone et al., 2020). Baume et al. found that methyltestosterone was metabolized into 17α -methyl-5 β -androstane-3 α, 17β -diol by gut flora and then eliminated from the body in urine; by contrast, the intestinal dysbacteriosis may result in methyltestosterone overaccumulation (Schweizer Grundisch et al., 2014). In accordance with the above speculation, the rats receiving fecal microbiota from PCOS individuals revealed a significant enhancement in serum T concentrations (Figure 5E). Therefore, these results suggested that gut microbiota dysbiosis may be a contributor to the elevation of serum androgens in PCOS patients.

Patients with PCOS were usually accompanied by chronic low-grade inflammation. Thereby, many studies believed that inflammation likely promoted occurrence and development of PCOS. Specifically, the activated pro-inflammatory signaling pathways phosphorylate the serine residues on insulin receptor substrate-1 (IRS-1) protein, and thus hamper its crosstalk with insulin receptor β subgroups (Hotamisligil et al., 1993, 1996). The dysfunction of signal transmission leads to the translocation of glucose transporter type 4 (GLUT4) and the disturbance of insulin signaling pathway, which further triggers insulin resistance and more insulin secretion (Hotamisligil et al., 1993, 1996). The excess insulin in ovarian tissue stimulates follicle theca cells to produce androgens (Nestler et al., 1998). Additionally, insulin also suppresses FSH production in granulosa cells, which thereby elevates LH/FSH. And the latter adversely affects the growth and development of follicles and induces polycystic ovary morphology (Adashi et al., 1981). In the present study, the great majority of microbial strains enriched in subjects with PCOS were pro-inflammatory microorganisms (Figure 1A). Functional analyses revealed that these pro-inflammatory microbial species over-expressed the enzyme MTOR and then stimulated multiple pro-inflammatory signaling pathways, including mTOR, ErbB, JAK–STAT, and PI3K-Akt signaling pathways in PCOS (Figure 2). Keeping in line with the above results, the FMT-conducted animal experiments demonstrated that the pro-inflammatory strains-enriched gut microbiota derived from PCOS patients significantly improved levels of serum inflammatory cytokine CRP and HOMA-IR in rats (Figure 5O). In previous studies, targeting PI3K/Akt/mTOR signaling pathways have been shown to ameliorate PCOS phenotypes (Chen et al., 2019, 2021; Liu et al., 2021). Additionally, we found that the strains elevated in PCOS also expressed the SREBP1 protein, by which, directly perturbing insulin signaling pathway and inducing insulin resistance (Figure 2). It was thereby plausible that gut microbiota might provide a novel therapeutical target for PCOS, which was supported by our FMT-conducted animal experiment (Figure 6).

In the present study, multiple strains including *F. prausnitzii*, *R. hominis*, *P. merdae*, *O. splanchnicus*, *P. succinatutens*, and *P. gordonii* had the lower abundance in PCOS when compared to healthy controls (Figure 1A), and these microorganisms were reported to be the potential beneficial microbiota, boosting host health. Consistent with our results, a recent study conducted by Chu et al. also showed a significant reduction in *F. prausnitzii* and *P. merdae* in patients with PCOS (Chu et al., 2020). *Faecalibacterium prausnitzii* seems a bright and promising strain due to its excellent performance in anti-inflammation and gut barrier maintenance (Quévrain et al., 2016; Lopez-Siles et al., 2017). A previous paper reported that *R. hominis* was able to promote intestinal melatonin production by stimulating the phosphorylated cAMP-response element-binding protein (p-CREB)-arylalkylamine N-acetyltransferase (AANAT) pathway (Song et al., 2021). Melatonin has been proved to alleviate PCOS phenotypes by various studies (Yu et al., 2019; Yi et al., 2020). Additionally, *F. prausnitzii* and *R. hominis* are the short-chain fatty acid producers, and SCFAs have many benefits including anti-inflammation, immunity booster, and metabolism improvement (Canfora et al., 2015; Dalile et al., 2019). Abundance of *F. prausnitzii* and *R. hominis* was reduced, suggesting that the production of SCFAs was suppressed in PCOS, which was supported by previous studies (Zhang et al., 2019; Łagowska and Drzymała-Czyż, 2022).

Almost all strains from the genus *Bacteroides* were decreased in the PCOS group (Figure 1A). Keeping in line with our results, Kelly et al. revealed a significant reduction in *Bacteroides* in letrozole-induced PCOS (Kelley et al., 2016). In this study, most of the species from *Bacteroides* showed strong negative associations with FSIns and HOMA-IR (Figure 1B). A recent study conducted by Tiffany et al. demonstrated that *Bacteroides* depletion was positively correlated with vascular dysfunction and glucose intolerance in patients with obesity (Trikha et al., 2021). Mechanistically, *Bacteroides* contains the genes synthesizing menaquinones (Vit K2), which modulate the endothelial function and blood pressure through a vitamin K2-dependent pathway (Walther et al., 2013; Liu et al., 2020). Also, the *Bacteroides* strains had a well-characterized pathway to produce folate (Qiao et al., 2020). It seemed thereby persuasive that the inhibited functions of gut microbiota producing folate, riboflavin and biotin in PCOS possibly resulted from the reduction in abundance of the family species from the genus *Bacteroides*, and then led to the lower contents of these vitamins (Supplementary Table S1). Coincidently, a targeted metabolomics study demonstrated that plasma concentrations of folate, biotin, and riboflavin were really downregulated in patients with PCOS when compared to healthy subjects (Szczuko et al., 2020).

Fecal metagenomics and serum metabolomics analyses demonstrated the inhibition in porphyrin and chlorophyll metabolisms in PCOS (Figure 4; Supplementary Figure S6). In microbiome, the intestinal microbiota revealed the lower expression of enzymes (phpB, hemZ, and cobD) involved in porphyrin and chlorophyll metabolism in PCOS than healthy subjects (Figure 2). In serum metabolome, bilirubin and 4E,15Z-bilirubin IXa that are the two metabolites of heme were downregulated in patients with PCOS (Supplementary Table S3). Bilirubin is a potent radical scavenger, which neutralizes reactive oxygen species (ROS) and regulates T helper type 17 immune responses (Hamoud et al., 2018). Besides, bilirubin activates peroxisome proliferator-activated receptor-α, which promotes the transport of fatty acids into mitochondrial matrix for β-oxidation, reducing fatty accumulation (Stec et al., 2016). Correlation analysis showed that bilirubin was significantly positively associated with *B. coprocola* and *B. massiliensis*, but negatively correlated with *A. pelogenes*, *A. xylosoxidans*, *V. fortis*, and *S. melioti* (Supplementary Figure S4).

By integrating intestinal metagenomics and serum metabolomics datasets, we found that seven strains (*S. meliloti*, *A. xylosoxidans*, *V. fortis*, *A. prlogenes*, *Porphyrobacter* sp. HL-46, *Pseudomonas* sp. M1, and *Leisingera* sp. ANG-Vp) illustrated the strong correlation with three metabolites [Ganglioside GM3, Cer(d16:2/22:0), and 3Z,6Z,9Z-Pentacosatriene; Figure 4A]. Ganglioside GM3 is a crucial component of the cytomembrane and is mainly expressed in mature and cystic follicles rather than primary and secondary follicles. According to previous studies, inflammatory responses stimulate production of ganglioside GM3, and over-accumulation of ganglioside GM3 aggravates the phosphorylation of granulosa cells (Hattori and Horiuchi, 1992b). As the above discussed, the phosphorylated granulosa cells result in the reduction in FSH secretion. Additionally, there is a bidirectional interaction between ganglioside GM3 and LH, wherein ganglioside GM3 could promote LH secretion, in return, LH has the capacity of boosting ganglioside GM3 biosynthesis (Hattori and Horiuchi, 1992a; Choo et al., 1995). Indeed, the modifications of gut microbiota by FMT could regulate levels of LH and FSH and ovarian histopathology in the rat trials (Figures 5, 6). However, whether gut microbiota modulates ovary functions by mediating ganglioside GM3 remains to need more investigation. Based on the single/multi-omics datasets, it was found that the gut microbiota-serum metabolites-based panel showed the highest predictivity (AUC:1.0) of PCOS, suggesting that these microbial and metabolic variables could be further developed as biomarkers for the diagnosis and treatment of this disorder (Figure 4D).

In summary, we undertook a multi-omics analysis of subjects with PCOS and integrated these data using systems methods to identify key features of this disorder. We showed the specific changes in the gut microbial compositions and their causing metabolic disturbances underlying PCOS. We also investigated the influence of these microbial changes on the host metabolism using serum metabolomics profiling, which was further confirmed by FMT. Herein, we revealed the potential molecular mechanisms underlying gut microbiota modulating pathogenesis of PCOS. We envisage that these findings may provide novel insights into discovering reliable predictive clinical biomarkers and developing efficient therapeutic strategies for PCOS.

## Data availability statement

TThe data presented in the study are deposited in the NCBI Sequence Read Archive database repository (https://www.ncbi.nlm.nih.gov/bioproject/PRJNA791492), accession number PRJNA791492.

## Ethics statement

The studies involving human participants were reviewed and approved by the ZhuJiang Hospital of Southern Medical University Institutional Review Board. The patients/participants provided their written informed consent to participate in this study. The animal study was reviewed and approved by the Institutional Animal Care and Use Committee of Sun Yat-sen University.

## Author contributions

All authors contributed to the study conception and design. ZY and HF were responsible for material preparation, sample collection, data analysis, animal experiments, and the first draft of the manuscript. HS and XC performed material preparation and metagenomic and metabolomic data collection. YW and JH carried out the clinical sample collection. The metabolomics profiling and revision of the previous versions of the manuscript were performed by YH. ZX and XW participated in the entire research progress, including data analysis and visualization and writing and revising the manuscript. All authors contributed to the article and approved the submitted version.

## Funding

This work was supported by the National Natural Science Foundation of China (no. U1903211 and 82174104), Guangdong Provincial Health Economics Association Scientific Research Project (no. 2021WJZD09), Innovation Training Project of Guangdong (nos. S202012121172 and S202112121087), Natural Science Foundation of Guangdong (no. 2019A1515010452), Guangdong Basic and Applied Basic Research Foundation (no. 2020A1515110406), and the GDAS′ Projection of Science and Technology Development (nos. 2021GDASYL-20210103037).

## Conflict of interest

The authors declare that the research was conducted in the absence of any commercial or financial relationships that could be construed as a potential conflict of interest.

## Publisher’s note

All claims expressed in this article are solely those of the authors and do not necessarily represent those of their affiliated organizations, or those of the publisher, the editors and the reviewers. Any product that may be evaluated in this article, or claim that may be made by its manufacturer, is not guaranteed or endorsed by the publisher.

## Footnotes

^1^ http://metlin.scripps.edu

^2^ http://www.hmdb.ca

^3^ http://www.lipidmaps.org

^4^ http://metflow.zhulab.cn/

^5^ https://www.metaboanalyst.ca/
