# Supplementary material for: Multi-omics analyses reveal the specific changes in gut metagenome and serum metabolome of patients with polycystic ovary syndrome
Source: Front Microbiol. 2022 Oct 19;13:1017147. doi: 10.3389/fmicb.2022.1017147 (PMC9627625; doi:10.3389/fmicb.2022.1017147)
Supplement: Supplementary file 2 [file Data_Sheet_2.pdf]

Supplementary Figure caption

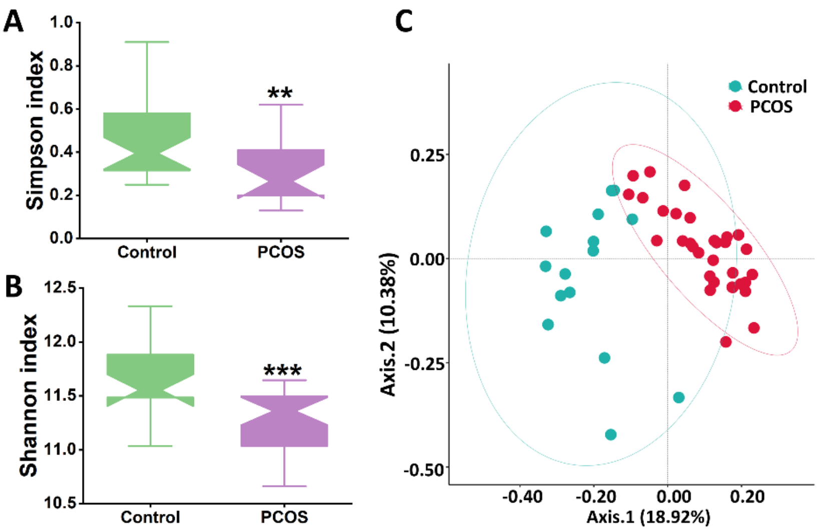

Figure S1: Gut microbial diversity in PCOS patients and healthy controls. Simpson index (A) and Shannon index (B) represent alpha-diversity. Bray_cruits based PCoA score plot (C) stands for beta-diversity. Differences of alpha-diversity (Simpson and Shannon indexes) are compared by the two-tailed Mann-Whitney U-test. Data are shown as mean ± s.d. ** $P < 0.01$, *** $P < 0.001$.

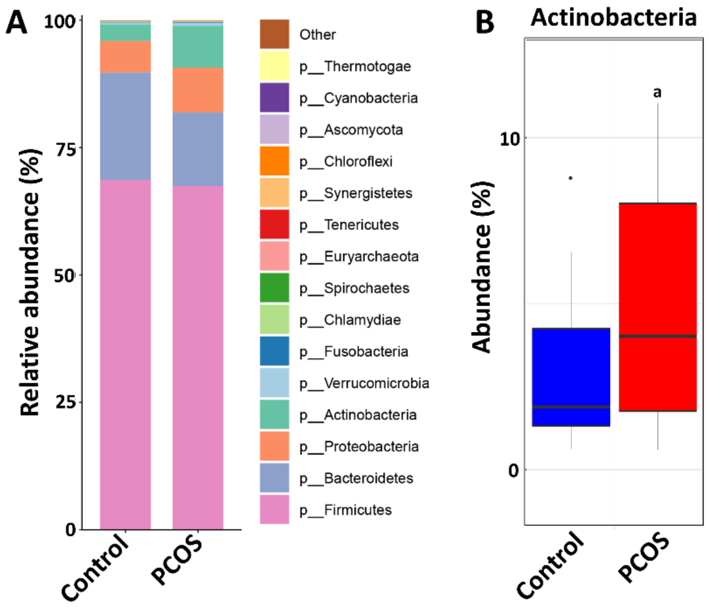

Figure S2: Bacterial phyla composition structure of PCOS and control groups. (A). The top fifteen abundant gut microbial composition between two groups. (B). Differential bacterial phyla with abundance of over 0.05% between PCOS and healthy controls. The comparison between two groups is performed by the two-tailed Mann-Whitney U-test, and P is adjusted by Benjamini-Hochberg method. Data are shown as mean ± s.d. ^{a} $P < 0.05$.

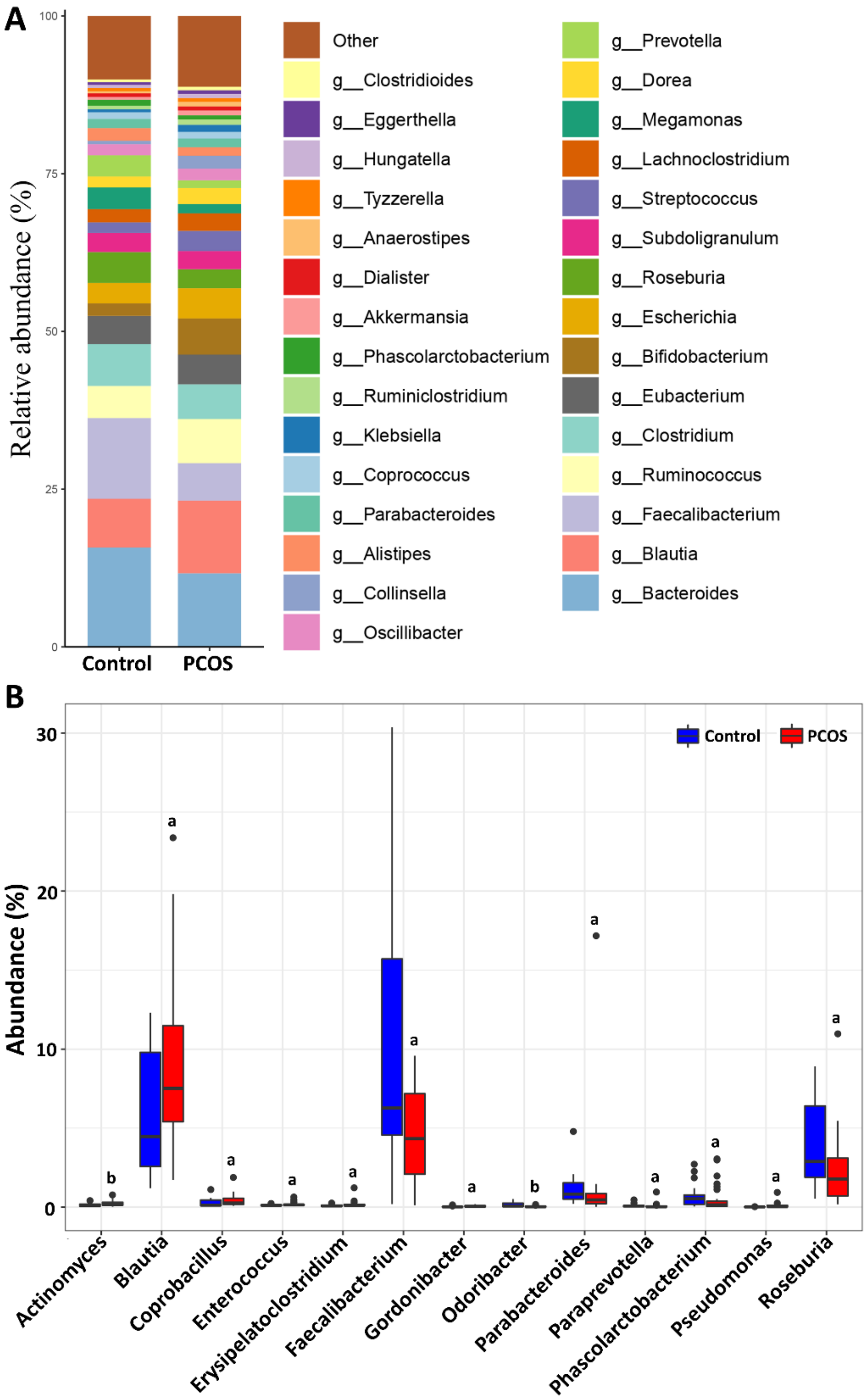

Figure S3: Microbial genus community composition of PCOS. (A). The top thirty abundant gut microbial genera between two groups. (B). Different microbial genera with abundance of over 0.05% between PCOS and healthy controls. The comparison between two groups is performed by the two-tailed Mann-Whitney U-test, and P value is adjusted by Benjamini-Hochberg method. Data are shown as mean ± s.d. [a] $P < 0.05$; [b] $P < 0.01$.

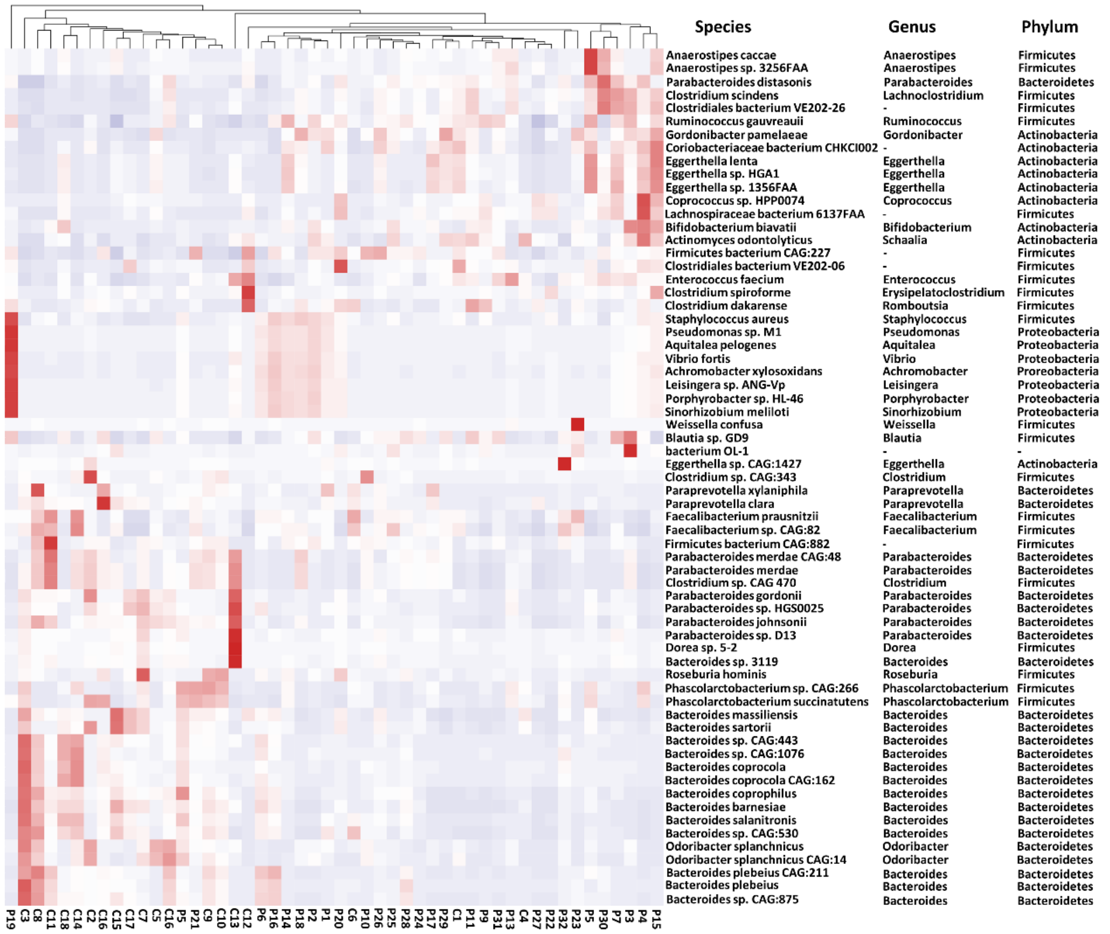

Figure S4: Distribution of differential gut microbial strains in each sample. The differential gut microbial species between PCOS patients and healthy participants are identified using LEfSe, and the threshold value of LDA score is set as less than 2.0, and *P* adjusted < 0.05.

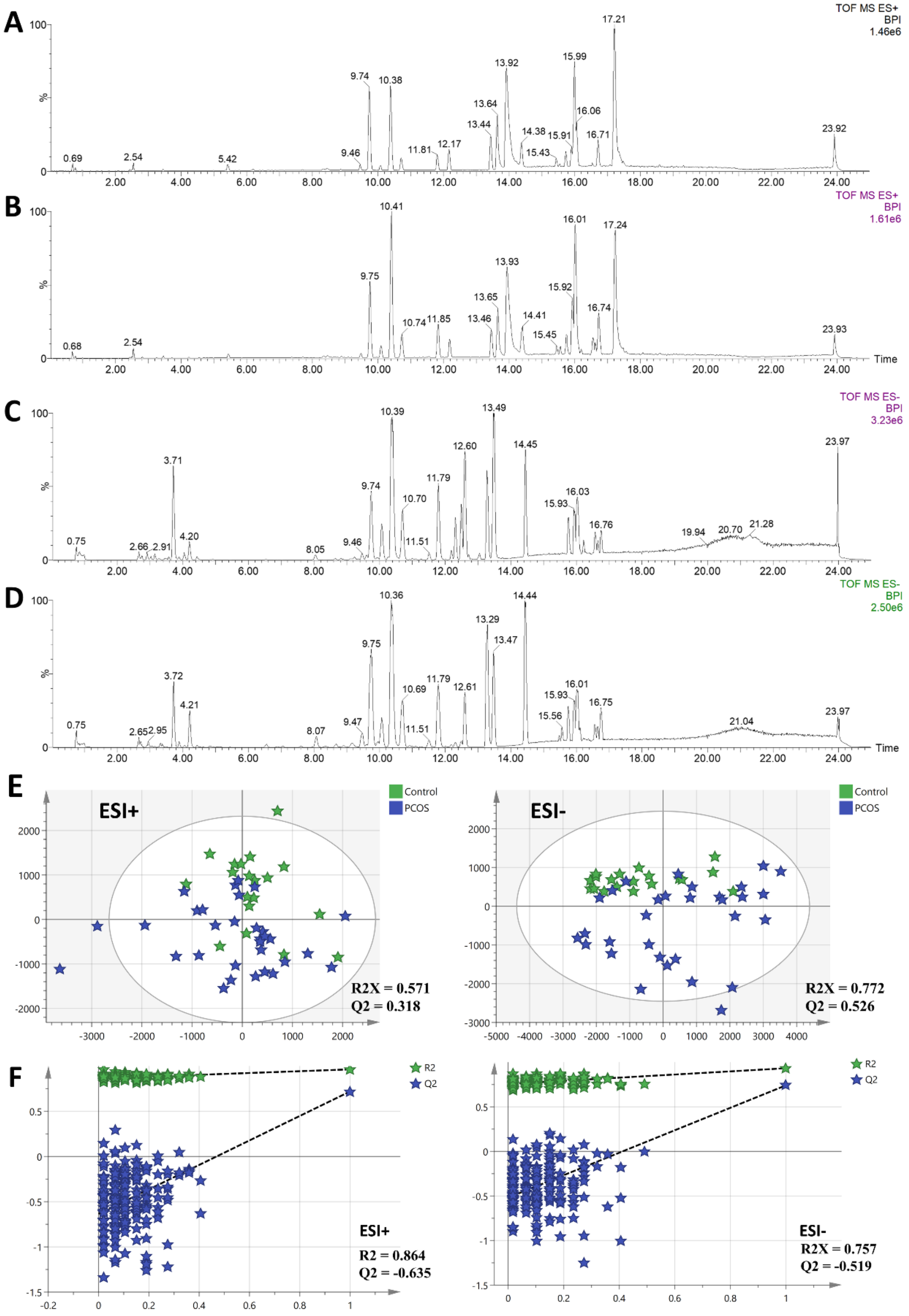

Figure S5: Serum metabolomics profiling of PCOS and healthy participants. The presentive base peak chromatogram of serum samples from PCOS and healthy subjects under positive (A, C) and negative (B, D) ion modes. (E) Principal component analysis (PCA) score plots are illustrated under positive (Left) and negative (Right) ion modes. (F) Permutation test plots (n = 200) are used to validate the reliability of the orthogonal partial least-squares discrimination analysis (OPLS-DA) models under positive (Left) and negative (Right) ion modes with cross validation CV-ANOVA P = 4.89E-8 and 1.13E-8, respectively.

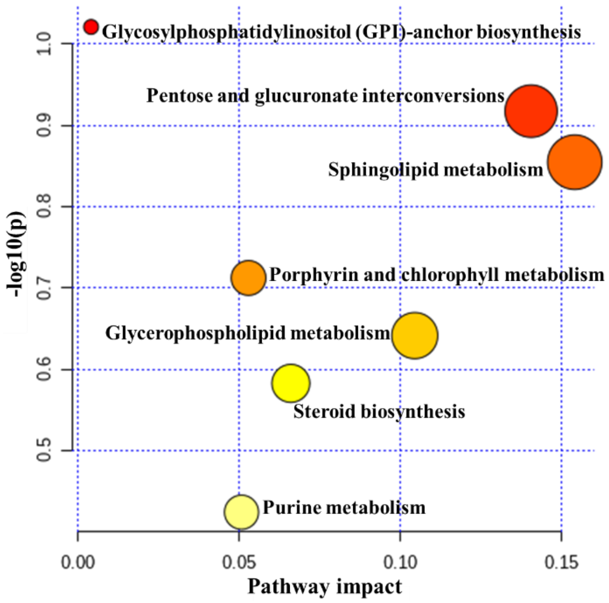

Figure S6: Pathway analysis is performed by MetaboAnalyst software using differential metabolites between PCOS and healthy individuals.

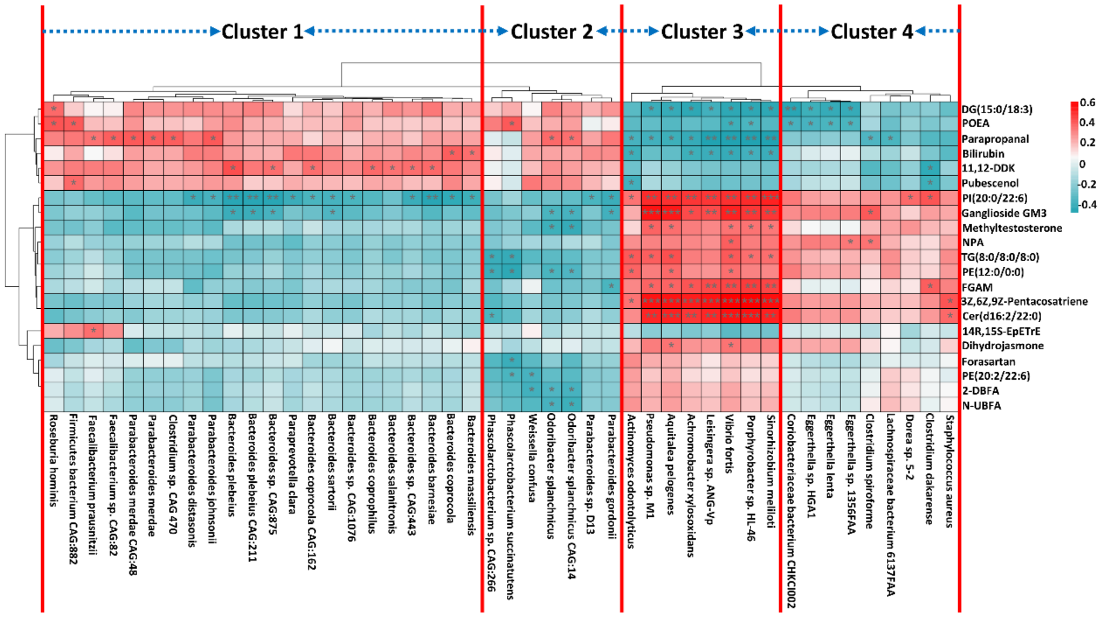

Figure S7. Integrative correlation heatmap analysis using metagenomics and metabolomics data. Significantly changed microbial strains are clustered into 4 groups. DG, diacylglycerol; POEA, palmitoleoyl ethanolamd; 11,12-DDK, 11,12-Dimethoxydihydrokawain; PI, phosphatidylinositols; NPA, N-[2-(3,4-dimethoxyphenyl)ethyl]-3-[4-methoxy-3-(sulfooxy)phenyl]propanimidic acid; TG, triglyceride; PE, phosphatidylethanolamine; FGAM, 5'-Phosphoribosyl-N-formylglycinamidine; Cer, ceramide; 14R,15S-EpETrE, 14,15-Epoxyeicosatrienoic acid; 2-DBFA, 2-Dodecylbenzenesulfonic acid; N-UBFA, N-Undecylbenzenesulfonic acid. Correlation coefficient between two parameters was calculated based on Pearson's approach. * $P < 0.05$, ** $P < 0.01$, *** $P < 0.001$.

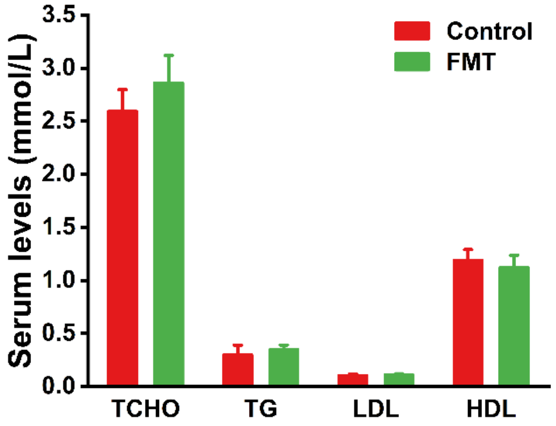

Figure S8: The effect of gut microbiota from PCOS patients on lipid metabolism in ABX-treated rats. TCHO, total cholesterol; TG, triglyceride; LDL, low-density lipoprotein; HDL, high-density lipoprotein. n = eight rats in each group. Data are shown as mean ± s.d. Difference is compared by one-way ANOVA followed by Tukey's post-hoc test.
